# Fenômeno de Chatterjee: O Coração se Lembra do que a Mente Esquece

**DOI:** 10.36660/abc.20250418

**Published:** 2026-03-04

**Authors:** Miguel Vicente, Eric Monteiro, Domingos Ramos, Lino Gonçalves

**Affiliations:** 1 Cardiology Department Coimbra Hospital and University Centre Coimbra Portugal Cardiology Department, Coimbra Hospital and University Centre, Coimbra – Portugal; 2 Cardiovascular and Thoracic Centre Clínica Girassol Luanda Angola Cardiovascular and Thoracic Centre – Clínica Girassol, Luanda – Angola

**Keywords:** Marca-Passo Artificial, Arritmias Cardíacas, Relatos de Casos

## Introdução

O fenômeno de Chatterjee, também conhecido como memória cardíaca (MC), é definido como alterações persistentes na onda T do eletrocardiograma após episódios de ritmos estimulados.^1^ Essas alterações se tornam evidentes quando o padrão normal de ativação ventricular é restabelecido, o que muitas vezes é confundido com condições patológicas como miocardite, hipertrofia e pré-excitação ventricular, distúrbios hidroeletrolíticos e hipertensão intracraniana, sendo o cenário mais grave a isquemia miocárdica aguda.^1 - 3^

Essa entidade nosológica, observada em cerca de um terço dos pacientes com marca-passos e com prevalência de aproximadamente 80% nos casos com alta porcentagem de estimulação ventricular, foi relatada pela primeira vez exatamente 110 anos atrás por White, que descreveu a inversão transitória da onda T (TWI) após extrassístoles ventriculares.^2^ Vinte e cinco anos depois, ondas T anormais de duração variável foram descritas após períodos de pré-excitação ventricular intermitente, síndrome pós-taquicardia ventricular e complexos ventriculares aumentados pela toxicidade de bloqueadores de canais de sódio.^4^

Em 1969, Kanu Chatterjee et al., em um estudo observacional, descreveram esses achados em 39 pacientes, denominando-os MC ou Fenômeno de Chatterjee.^5^ Rosenbaum et al. propuseram a primeira hipótese de como a ativação ventricular anormal poderia gerar alterações na onda T como consequência de um mecanismo gerado por modulação elétrica (através de dispositivos eletrônicos cardíacos implantáveis).^6^

Chatterjee et al. também demonstraram que a duração da inversão da onda T é diretamente proporcional à duração da estimulação elétrica (quando estimulada por 10 minutos ou 2 anos, por exemplo, a onda T permanece invertida por 10 minutos e aproximadamente 18 meses, respectivamente).^7^

Os autores relatam o caso de uma mulher de 85 anos que recebeu um diagnóstico precoce de MC, o que evitou estratificação invasiva desnecessária.

## Caso

**Figura 1:**
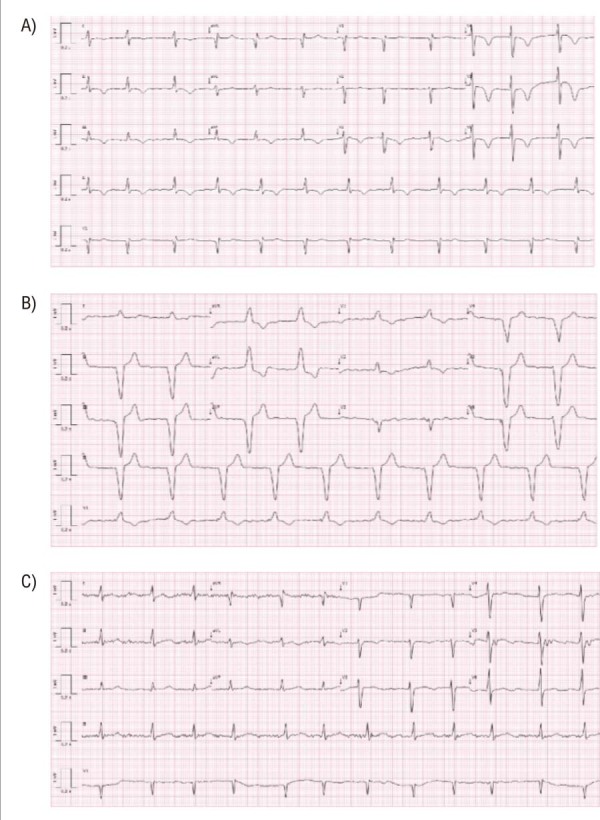
– A) ECG: Fibrilação atrial, inversão da onda T em DII, DIII, aVF, V3 a V6, Memória cardíaca. B) O ECG obtido durante a estimulação do ventrículo direito pelo marca-passo mostrou complexos QRS estimulados negativos nas derivações que apresentaram novas inversões da onda T (DII, DIII, aVF e V3–V6). C) ECG antes da implantação do marca-passo, ritmo de fibrilação atrial com resposta ventricular controlada.

Paciente de 85 anos com histórico de implante de marca-passo devido à síndrome de taquicardia-bradicardia e episódios recorrentes de síncope. Duas semanas após o implante, foi encaminhada ao pronto-socorro se queixando de dor torácica aguda e um tanto opressiva, sem irradiação, além de sensação de falta de ar e prostração há dois dias. Com teste positivo para COVID-19, iniciou tratamento direcionado e permaneceu hemodinamicamente estável. O eletrocardiograma revelou fibrilação atrial com inversões difusas da onda T nas derivações DII, DIII, aVF e V3-V6 ( Figura 1A ).

O teste seriado de troponina cardíaca ultrassensível apresentou resultado negativo. Foi solicitada uma avaliação cardiológica para descartar um evento isquêmico coronariano agudo. A análise do dispositivo revelou modo VVI, com 72% de estimulação ventricular e um complexo QRS alargado (199 ms). O limiar de estimulação foi de 0,5 ms, a sensibilidade de 2,5 mV e a impedância de 684 Ω, todos dentro dos limites normais. Observou-se um complexo QRS estimulado com polaridade negativa nas derivações que apresentaram nova inversão da onda T ( Figura 1B ). No eletrocardiograma pré-implante, o ritmo básico era fibrilação atrial com resposta ventricular controlada ( Figura 1C ), e observou-se que as alterações de corrente não correspondiam a um evento isquêmico, mas sim ao fenômeno de Chatterjee ou MC.

## Discussão

A MC é uma condição que pode causar confusão no diagnóstico, especialmente entre médicos menos experientes. Quando associada a uma apresentação clínica sugestiva, pode ser facilmente confundida com isquemia aguda, que geralmente requer tratamento invasivo imediato. Portanto, os profissionais de emergência devem saber como identificá-la ou descartá-la corretamente para evitar exames desnecessários e fornecer atendimento eficiente aos pacientes.^1 , 7 , 8^

Embora a MC seja considerada um achado benigno, ela tem sido associada a um estado pró-arrítmico, especialmente em pacientes com intervalos QT longos, devido ao alto risco de Torsades de Pointes.^7 , 8^

Sua fisiopatologia ainda é objeto de muitas teorias, sendo a mais aceita a do estiramento mecânico e da tensão dos cardiomiócitos, resultantes da ativação artificial contínua, com consequente síntese e liberação de angiotensina II, que regula a corrente transitória de potássio externa (Ito) e a expressão de canais de potássio na superfície celular, diminuindo sua saída do interior da célula e aumentando o gradiente de repolarização ventricular. Subsequentemente, ocorre a internalização do receptor de angiotensina-1 e de uma subunidade do canal de potássio na superfície celular, levando à sua degradação e reduzindo a densidade de seus canais funcionais na corrente. Portanto, interrupções nesse processo levarão, compreensivelmente, a alterações na morfologia da onda T, fazendo com que ela permaneça após a estimulação por um certo período de tempo.^7 , 8^ Além disso, essas alterações na repolarização promovem a heterogeneidade da ativação elétrica cardíaca, causando dissincronia cardíaca.^2 , 8^

Certos critérios ajudam a diferenciar a TWI na isquemia cardíaca da MC. Na isquemia da artéria descendente anterior, a TWI apresenta um eixo dextrogiro no plano frontal, com inversão nas derivações DI e aVL. Em casos raros de isquemia da artéria coronária direita, que causa TWI precordial com eixo levógiro, as inversões são mais profundas em DII, DIII e aVF do que nas derivações precordiais. A estimulação apical do ventrículo direito e do ramo esquerdo do feixe de His gera vetores QRS positivos em DI e aVL, produzindo ondas T positivas nessas derivações e inversões mais profundas nas derivações precordiais em comparação com as derivações inferiores durante a retomada da condução normal^2 , 8^ (MC). No paciente em questão, o posicionamento do cateter eletrodo no ápice do ventrículo direito é a base para a morfologia QRS positiva observada em V1 e V2.

Com base no quadro clínico, sua evolução e nos resultados negativos da ultrassonografia cardíaca seriada, se concluiu que os sintomas estavam relacionados à infecção por COVID-19. Além disso, as alterações no eletrocardiograma não indicaram a ocorrência de um evento coronariano agudo. Consequentemente, o paciente recebeu alta da cardiologia.

O diagnóstico precoce desse fenômeno evitou a necessidade de exames e procedimentos invasivos desnecessários diante de uma alteração eletrocardiográfica sugestiva. O reconhecimento dessa alteração eletrocardiográfica resultou na redução de custos, exames desnecessários, procedimentos invasivos e seus riscos, permitindo o tratamento seguro do paciente.

## Conclusão

O fenômeno da Memória cardíaca ou de Chatterjee deve ser reconhecido precocemente para evitar abordagens invasivas desnecessárias.

## References

[1] Shvilkin A, Huang HD, Josephson ME (2015). Cardiac Memory: Diagnostic Tool in the Making. Circ Arrhythm Electrophysiol.

[2] Grimm W, Luck K, Greene B, Parahuleva M (2019). Cardiac Memory Following Pacemaker Implantation. Herzschrittmacherther Elektrophysiol.

[3] White P (1915). Alteration of the Pulse: A Common Clinical Condition. Am J Med Sci.

[4] Campbell M (1942). Inversion of t Waves after Long Paroxysms of Tachycardia. Br Heart J.

[5] Chatterjee K, Harris A, Davies G, Leatham A (1969). Electrocardiographic Changes Subsequent to Artificial Ventricular Depolarization. Br Heart J.

[6] Rosenbaum MB, Blanco HH, Elizari MV, Lázzari JO, Davidenko JM (1982). Electrotonic Modulation of the T Wave and Cardiac Memory. Am J Cardiol.

[7] Tafoya C, Singh A (2019). Cardiac Memory: A Case Report and Review of the Literature. J Emerg Med.

[8] Viskin S, Chorin E, Schwartz AL, Kukla P, Rosso R (2022). Arrhythmogenic Effects of Cardiac Memory. Circulation.

