# Supplementary material for: Fenômeno de Chatterjee: O Coração se Lembra do que a Mente Esquece
Source: Arq Bras Cardiol. 2026 Mar 4;123(2):e20250418. [Article in Portuguese] doi: 10.36660/abc.20250418 (PMC13128189; doi:10.36660/abc.20250418)
Supplement: Translation

# Translation: Chatterjee Phenomenon: The Heart Remembers What the Mind Forgets

**DOI:** 10.36660/abc.20250418i

**Authors:** Miguel Vicente, Eric Monteiro, Domingos Ramos, Lino Gonçalves

**Affiliations:** 1 Portugal Cardiology Department, Coimbra Hospital and University Centre, Coimbra – Portugal; 2 Angola Cardiovascular and Thoracic Centre – Clínica Girassol, Luanda – Angola

**Keywords:** Artificial Pacemaker, Cardiac Arrhythmias, Case Reports

## Introduction

The Chatterjee phenomenon, also known as cardiac memory (CM), is defined as persistent changes in the T wave on the electrocardiogram after episodes of stimulated rhythms.^1^These changes become evident when the normal pattern of ventricular activation is reestablished, which is often confused with pathological conditions such as myocarditis, hypertrophy, and ventricular pre-excitation, hydroelectrolytic disorders, and intracranial hypertension, where the most serious scenario is acute myocardial ischemia.^1 - 3^

This nosological entity, observed in about one-third of patients with pacemakers and with a prevalence of around 80% in cases with a high percentage of ventricular stimulation, was first reported exactly 110 years ago by White, who described transient T wave inversion (TWI) after premature ventricular beats.^2^Twenty-five years later, abnormal T waves of varying duration were described after periods of intermittent ventricular pre-excitation, post-ventricular tachycardia syndrome, and ventricular complexes augmented by sodium channel blocker toxicity.^4^

In 1969, Kanu Chatterjee et al., in an observational study, described these findings in 39 patients, calling them CM or the Chatterjee phenomenon.^5^Rosenbaum et al. proposed the first hypothesis of how abnormal ventricular activation could generate changes in the T wave as a consequence of a mechanism generated by electrical modulation (through implantable cardiac electronic devices).^6^

Chatterjee et al. also showed that the duration of TWI is directly proportional to the duration of electrical stimulation (when stimulated for 10 minutes or 2 years, for example, the T wave remains inverted for 10 minutes and approximately 18 months, respectively).^7^

The authors report the case of an 85-year-old woman who had an early diagnosis of CM, which avoided unnecessary invasive stratification.

## Case

An 85-year-old patient with a history of pacemaker implantation due to tachycardia-bradycardia syndrome and recurrent episodes of syncope. Two weeks after implantation, she was referred to the emergency department, complaining of sharp, somewhat oppressive chest pain without irradiation and a feeling of breathlessness and prostration for two days. Having tested positive for COVID-19, she began targeted treatment and remained hemodynamically stable. The electrocardiogram revealed atrial fibrillation with diffused T-wave inversions in leads DII, DIII, aVF, and V3-V6 ( Figure 1A ).

**Figure 1:** – A) ECG: Atrial fibrillation, T wave inversion in DII, DIII, aVF, V3 to V6, Cardiac memory. B) ECG obtained during right ventricular pacing by the pacemaker showed stimulated QRS complexes negative in leads that exhibited new T wave inversions (DII, DIII, aVF, and V3–V6). C) ECG before pacemaker implantation, atrial fibrillation rhythm with controlled ventricular response.

The serial ultrasensitive cardiac troponin test was negative. A cardiology assessment was requested to rule out an acute coronary ischemic event. Interrogation of the device revealed VVI mode, with 72% ventricular pacing and a widened QRS (199 ms). The pacing threshold was 0.5 ms, sensitivity was 2.5 mV, and impedance was 684 Ω, all within normal limits. Stimulated QRS with negative polarity was observed in the leads that showed new TWI. ( Figure 1B ). In the pre-implantation electrocardiogram, the basic rhythm was atrial fibrillation with controlled ventricular response ( Figure 1C ), and it was noted that the current alterations did not correspond to an ischemic event, but rather to the Chatterjee phenomenon or CM.

## Discussion

CM is a condition that can cause confusion in diagnosis, especially among less experienced doctors. When associated with a suggestive clinical presentation, it can easily be confused with acute ischemia, which usually requires immediate invasive treatment. Therefore, emergency professionals must know how to correctly identify or rule it out to avoid unnecessary tests and provide efficient care to patients.^1 , 7 , 8^

Although CM is believed to be a benign finding, it has been associated with a proarrhythmic status, especially in patients with long QT intervals, due to the high risk of *Torsades de Pointes* .^7 , 8^

Its pathophysiology is still the subject of many theories, the most widely accepted being that of mechanical stretching and tension of cardiomyocytes, resulting from continuous artificial activation, with consequent synthesis and release of angiotensin II, which regulates the external transient potassium current (Ito) and the expression of potassium channels on the cell surface, decreasing its output from inside the cell and increasing the ventricular repolarization gradient. Subsequently, there is internalization of the angiotensin-1 receptor and a subunit of the potassium channel on the cell surface, leading to its degradation and reducing the density of its functional channels in the current. Therefore, interruptions in this process will understandably lead to changes in the morphology of the T wave, causing it to remain after stimulation for a certain period of time.^7 , 8^In addition, these changes in repolarization promote heterogeneity of cardiac electrical activation, causing cardiac dyssynchrony.^2 , 8^

Certain criteria help differentiate TWI in cardiac ischemia from memory. In anterior descending artery ischemia, TWI has a right axis in the frontal plane, with inversion in leads DI and aVL. In rare cases of right coronary artery ischemia, which causes precordial TWI with a left axis, the inversions are deeper in DII, DIII, and aVF than in the precordial leads. Apical stimulation of the right ventricle and left bundle branch generates positive QRS vectors in DI and aVL, producing positive T waves in these leads and deeper inversions in the precordial leads compared to the inferior leads during the resumption of normal conduction^2 , 8^(CM). In the patient in question, the positioning of the electrode catheter at the apex of the right ventricle is the basis for the positive QRS morphology observed in V1 and V2.

Based on the clinical picture, its evolution, and negative serial ultrasensitive cardiac enzymology, it was concluded that the symptoms were related to COVID-19 infection. In addition, the changes in the electrocardiogram did not indicate the occurrence of an acute coronary event. As a result, the patient was discharged from cardiology.

The early diagnosis of this phenomenon avoided the need for unnecessary invasive tests and procedures in the face of a suggestive electrocardiographic change. Recognition of this electrocardiographic change resulted in reduced costs, unnecessary tests, invasive procedures, and their risks, allowing for safe treatment of the patient.

## Conclusion

The Cardiac memory or Chatterjee phenomenon should be recognized early to avoid unnecessary invasive approaches.
